# Driving Cessation and Social Participation in Late Life: A Systematic Review

**DOI:** 10.1093/geroni/igaa057.1504

**Published:** 2020-12-16

**Authors:** Weidi Qin

**Affiliations:** Case Western Reserve University, Cleveland, Ohio, United States

## Abstract

Driving cessation is a major life transition in late life, and can affect the quality of social life in older adults. The present study aims to systematically review the literature on how driving cessation affects social participation among older adults in the US. The study selection followed the Preferred Reporting Items for Systematic Reviews and Meta-Analyses (PRISMA). Extant literature published from 1990 to 2019 that examined driving cessation and social participation or social engagement among older adults in the US was searched using eight search engines: PsycINFO, CINAHL, SocIndex, AgeLine, MedLine, Scopus, Transportation Research Board Publication Index, and Cochrane Library. Quantitative studies that met the inclusion criteria were reviewed. The assessment of methodological quality was also conducted for included studies. In total, seven studies met the inclusion criteria. Six of the included studies found significant relationships between driving cessation and at least one domain of social participation, such as volunteering, employment, leisure-time activities, and the frequency of contacts. However, the measures of social participation were inconsistent across studies, which might explain that no effects of driving cessation were found on the structure of social network, such as contacts with friends and extended family. There is a need to assist older adults in successfully transitioning to driving cessation and maintaining the social participation. The overall quality of included studies is moderate based on the assessment of risk of bias and confounding.

